# Macrophage Migration Is Impaired within *Candida albicans* Biofilms

**DOI:** 10.3390/jof3030031

**Published:** 2017-06-22

**Authors:** Maria F. Alonso, Neil A. R. Gow, Lars P. Erwig, Judith M. Bain

**Affiliations:** Medical Research Council Centre for Medical Mycology, University of Aberdeen, Aberdeen AB25 2ZD, UK; m.f.alonso@abdn.ac.uk (M.F.A.); n.gow@abdn.ac.uk (N.A.R.G.); l.p.erwig@abdn.ac.uk (L.P.E)

**Keywords:** Candida, biofilm, macrophage, phagocytosis, migration, cell wall

## Abstract

*Candida albicans* is an opportunistic fungal pathogen that infects immunocompromised patients. Infection control requires phagocytosis by innate immune cells, including macrophages. Migration towards, and subsequent recognition of, *C. albicans* fungal cell wall components by macrophages is critical for phagocytosis. Using live-cell imaging of phagocytosis, the macrophage cell line J774.1 showed enhanced movement in response to *C. albicans* cell wall mutants, particularly during the first 30 min, irrespective of the infection ratio. However, phagocyte migration was reduced up to 2-fold within a *C. albicans* biofilm compared to planktonic fungal cells. Biofilms formed from *C. albicans* glycosylation mutant cells also inhibited macrophage migration to a similar extent as wildtype *Candida* biofilms, suggesting that the physical structure of the biofilm, rather than polysaccharide matrix composition, may hamper phagocyte migration. These data illustrate differential macrophage migratory capacities, dependent upon the form of *C. albicans* encountered. Impaired migration of macrophages within a *C. albicans* biofilm may contribute to the recalcitrant nature of clinical infections in which biofilm formation occurs.

## 1. Introduction

Human mucosal surfaces are frequently colonised by *Candida albicans*, although host surveillance by host innate defense normally prevents the transition from commensal colonisation to infection [1]. Protection against candidiasis relies mainly upon professional phagocytes, including neutrophils and macrophages, whose phagocytic uptake and degradation of the target microbe are critical to the inactivation of fungal particles [2]. *C. albicans* is a constituent of the commensal microflora, but can overgrow in the absence of normal control measures, including intact epithelial barriers, a competitive bacterial microbiota, and normal immune function. Fungal growth can occur within a diverse range of host compartments with varying pH and nutrient availabilities, to which this species readily adapts, resulting in multiple *C. albicans* morphologies that are differentially recognised by immune surveillance mechanisms [3,4,5,6]. In addition, fungal pathogens can alter and manipulate the environment in which they are growing by creating a biofilm.

Individual yeast cells can initiate biofilm formation on the surfaces of medical and prosthetic devices. *C. albicans* biofilms are characterised primarily by a supporting layer of yeast cells, from which filamentous hyphae and pseudohyphae are formed, and the secretion of extracellular material containing significant amounts of β-glucan and DNA [7]. *Candida* biofilms are recalcitrant to antifungal therapy, and can cause prosthetic devices to fail through biofouling of mechanisms. They also resist host immune defenses, and are a reservoir from which subsequent infections are disseminated [8]. The mortality rate for blood stream infections of *Candida* is elevated when biofilms form efficiently [9]. The response of immune cells to fungal biofilms has been the subject of little investigation, yet interactions between phagocytes and *C. albicans* will have a profound influence on the outcome of a fungal infection [2].

We have previously investigated the phagocytic interactions of individual *C. albicans* cells using live cell imaging and analysis methods, including tracking the individual migratory pathways of host cells [10,11,12]. This study compares real time interactions between host and pathogen to determine how the fungus *C. albicans* influences the migration activity of macrophages responding to individual fungal particles or 3D biofilm structures, thereby representing different fungal growth forms that may be encountered in the host. Phagocyte migration (mean track velocity) increased in response to individual *C. albicans* cells displaying altered cell wall architecture, confirming previous findings [10]. Cell wall mutants were selected that represent defects in *O*-mannosylation, *N*-mannosylation, *O*- and *N*-mannosylation, and phoshomannan (Table 1). Macrophage migration kinetics were markedly impaired (up to 2-fold) in a fungal biofilm, underlining that biofilms not only impair chemotherapeutic treatments, but also the effectiveness of the innate immune response. The approach used here offers valuable insight into the physical responses of professional phagocytes, within the clinically relevant context of 3D biofilms, at the host–pathogen interface.

## 2. Materials and Methods

### 2.1. C. albicans Strains and Growth Conditions

Glycosylation mutants used in this study (Table 1) were previously constructed in the *C. albicans* CAI4 serotype A background by targeted gene disruption, using *URA3* as a selectable marker [18]. A double mutant, the *mnt1*Δ*mnt2*Δ *O*-glycosylation mutant, was studied rather than individual mutants of *MNT1* or *MNT2*, as there is some degree of redundancy in the encoded mannosyltransferases [14]. A control strain was used in which a single reintegrated copy of the deleted *MNT1* gene was re-introduced, which regenerated the heterozygous genotype and restored *O*-mannosylation of cell wall proteins [14]. This served as an additional control, alongside the parental strain CAI4. All strains had been transformed with the integrating vector CIp10 to control for uniform expression of *URA3* [19] as to confer isogenic Ura status. *C. albicans* strains from glycerol stocks stored at −80 °C were grown in SC-Ura medium (6.9 g yeast nitrogen base without amino acids (Formedium, Norfolk, UK), 1 mL 1 M NaOH (BDH Chemicals, VWR International, Leicestershire, UK), 10 mL 1% (*w*/*v*) adenine hemisulphate salt (Sigma, Dorset, UK), 50 mL 40% d-glucose (Fisher Scientific, Leicestershire, UK), and 50 mL 4% SC-Ura dropout (Formedium), and were made up to 1000 mL in distilled H_2_O, with or without 2% (*w*/*v*) technical agar (Oxoid, Cambridge, UK)). Plates were incubated at 30 °C for 48 h and then stored at 5 °C. Liquid cultures were grown with continuous shaking at 200 rpm, at 30 °C. Biofilms were prepared by seeding µ-Slide 8-well chambers (ibidi GmbH, Munich, Germany) with 3 × 10^5^ Leicestershire live *C. albicans* yeast in Dulbecco’s Modified Eagle Medium (DMEM; Lonza, Slough, UK), supplemented with 10% (*v*/*v*) heat-inactivated FCS (Biosera, Ringmer, UK), 200 U/mL penicillin/streptomycin antibiotics (Invitrogen, Paisley, UK), and 2 mM L-glutamine (Invitrogen, Paisley, UK), at 37 °C for 48 h. There were two assay designs, with planktonic yeast added to either imaging dishes containing macrophages, or to fungal biofilms with macrophages added in.

### 2.2. Preparation of J774.1 Murine Macrophage Cell Line

J774.1 macrophages (ECACC, HPA, Salisbury, UK) were retrieved from stocks stored in liquid nitrogen. Cells were maintained in tissue culture flasks, in supplemented DMEM medium, at 37 °C, with 5% CO_2_. For phagocytosis assays, 1 × 10^5^ J774.1 macrophages, in 300 µL supplemented DMEM medium, were seeded onto μ-Slide 8-well chambers, and cultured overnight at 37 °C, with 5% CO_2_.

### 2.3. Macrophage Migration Assays

Live phagocytosis assays were prepared as described in earlier work [10,11,12,20,21]. Single *C. albicans* colonies from plates were cultured overnight in 5 mL SC-Ura medium as described above. On the day of the experiment, cells were harvested by centrifugation (6000 rpm, 1 min), washed three times with 1 mL phosphate buffered saline (PBS), resuspended in 1 mL PBS, and quantified using an Improved Neubauer haemocytometer chamber. Live imaging assays were conducted with 3 × 10^5^ yeast cells, combined with 1 × 10^5^ J774.1 macrophages, in supplemented DMEM, within μ-Slide 8-well chambers. Wells with fresh supplemented DMEM, but without fungi, were used as controls. Imaging was performed using an UltraVIEW VoX 3D spinning disk confocal microscope, using a 20× or 40× objective with Volocity 6.3 software (PerkinElmer, Cambridge, UK), and an environmental control chamber set at 37 °C, and 5% CO_2_. Images were captured using multipoint acquisition at 90 s intervals for 1 h, following a 15 min period to set up the microscope, and allow *C. albicans* cells to descend towards adherent macrophages. For some experiments, the multiplicity of infection (MOI) was changed so that 1 × 10^5^, 3 × 10^5^, or 10 × 10^5^ yeast cells were added to 1 × 10^5^ J774.1 macrophages in μ-Slide 8-well chambers immediately prior to imaging. Prior to biofilm 3D microscopy, fungal cell walls were stained with 5 µg·mL^−1^ Calcoflour White (CFW), and macrophages were stained with LysoTracker Red DND-99 (ThermoFisher), at a dilution of 1:1000 for 10 min prior to washing with DMEM. Macrophages were resuspended by gentle scraping, then washed and counted. Samples of 1 × 10^5^ macrophages were added to each well for biofilm and planktonic cell experiments. Three-dimensional imaging was conducted at 2 min intervals, using 561 and 405 laser lines to collect z-stacks of 60 µm thickness imaged at 1 µm intervals, following a 45 min period prior to image acquisition, to set up the imaging parameters and to allow for macrophages to descend.

### 2.4. Analysis of Live Cell Microscopy Movies

Volocity imaging and analysis software was used to analyse the live cell movies obtained. Three independent experiments were conducted for each condition, and within each experiment, duplicates were conducted for all conditions. 50 or 25 macrophages were randomly selected from each experimental condition (cell wall mutant or MOI migration, respectively), and were manually tracked at 90 s intervals for 1 h. Manual tracking of the migration process towards fungal particles allowed the construction of macrophage tracking diagrams, illustrating the distance travelled, directionality, and velocity, for each individual macrophage tracked. Quantitative migration data were analysed in 10 min bins to evaluate whether macrophage migration exhibited temporal variation over the time course. For 3D imaging, analysis of tracking at 2 min intervals was performed for the first 30 min of z-stack acquisition, beyond which photo-bleaching and toxicity were deemed to be potentially compromising. Mean velocities for each experimental condition were calculated and presented as values relative to the velocity of macrophages in wells with no *Candida*, or relative to relevant planktonic controls for biofilm experiments.

### 2.5. Statistical Analyses

Mean values and standard deviations were calculated. Statistical significance was determined by two-way analysis of variance (ANOVA), the categories being biological replicate and fungal strain, with Bonferroni Multiple Analysis Comparison Tests, or by one-way ANOVA and Tukey Multiple Analysis Comparison Tests. For comparisons of two groups, Student’s *t*-test was performed.

## 3. Results

### 3.1. Macrophage Migration towards C. albicans Is Not Altered by Multiplicity of Infection

**Figure 1:**
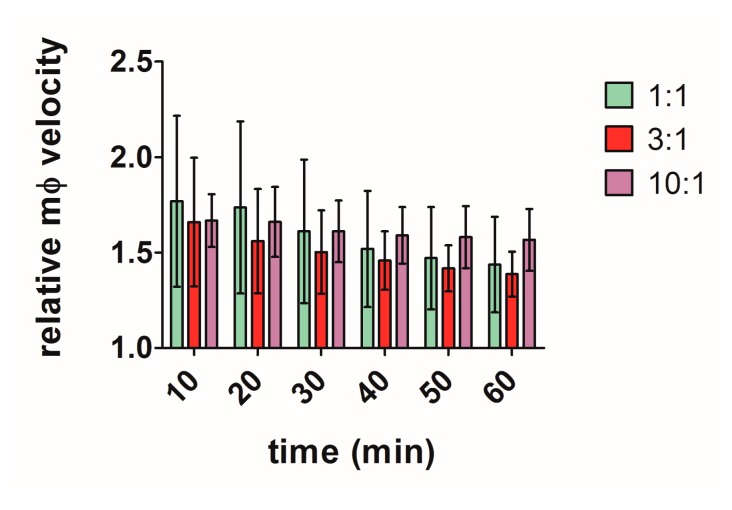
Quantitative analysis of macrophage migration in the presence of different multiplicity of infection (MOI) ratios of wildtype *C. albicans*. The chart shows mean relative macrophage velocity (relative to macrophage velocity without *Candida*) + SD of J774.1 macrophages when cultured with 1:1, 3:1 and 10:1 *C. albicans* ratios. Statistical significance was evaluated using two-way analysis of variance (ANOVA) and Bonferroni Multiple Analysis Comparison Tests. No statistically significant difference was observed in 25 macrophages analysed from independent experiments (*n* = 3).

We first characterised in detail the macrophage response to live *C. albicans*. Live cell video microscopy (a representative movie can be viewed: supplementary Video 1) combined with image analysis tools allowed tracking of individual cells. Representative images showing the tracking process of a single macrophage migrating towards, and finally engulfing, a *C. albicans* yeast cell are shown in Figure S1. Macrophages exhibited some degree of random movement, but were capable of directing their movement towards the *C. albicans* yeast cell target. J774.1 macrophages were exposed to live yeast *C. albicans* for up to 60 min, and time lapse captured at 90 s intervals. To elucidate macrophage migration dynamics towards increasing quantities of *C. albicans* cells, experiments were carried out using different MOI ratios (1:1, 3:1 and 10:1) of control strain CAI4. Tracking diagrams did not reveal any major differences between experiments carried out at these MOI ratios (not shown). Quantitative analysis revealed that no significant difference in migratory activity was observed between the experiments using the different MOI ratios throughout the 1 h observation period (Figure 1). Migration speed was greatest at earlier stages of the experiment and gradually decreased over time (Figure 1).

### 3.2. Macrophage Migration Dynamics towards C. albicans Is Affected by Fungal Cell Wall Composition

**Figure 2:**
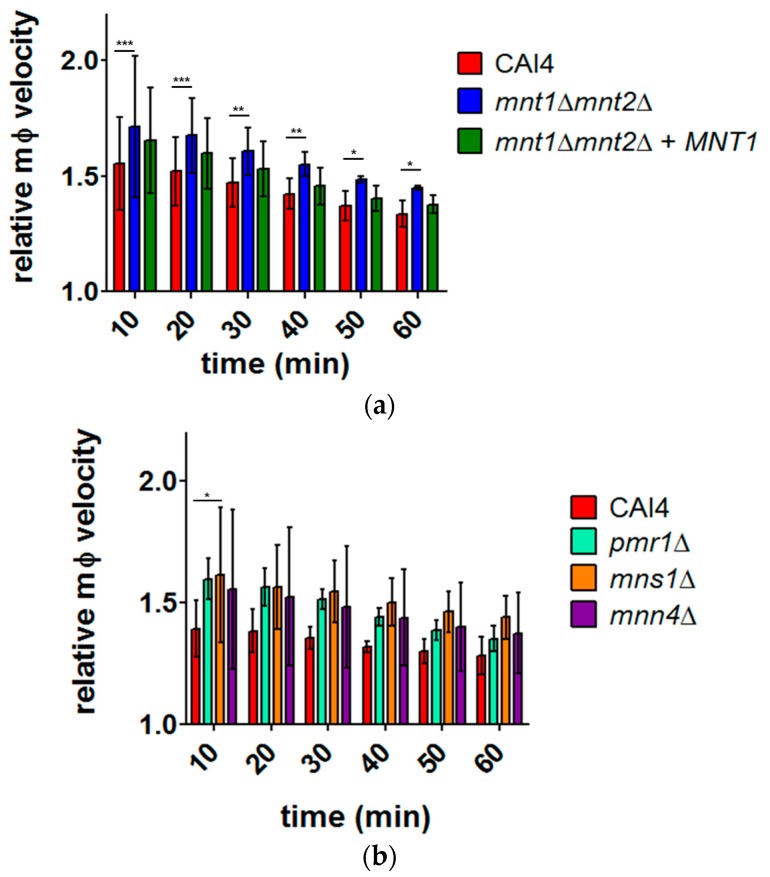
Quantitative analysis of macrophage migration towards wildtype and glycosylation *C. albicans* mutants. The charts show mean relative macrophage velocity (relative to macrophage velocity without *Candida*) + SD of J774.1 macrophages when cultured with: (**a**) CAI4, *mnt1*Δ *mnt2*Δ, and *mnt1*Δ *mnt2*Δ+*MNT1*; and (**b**) CAI4, *pmr1*Δ, *mns1*Δ, and *mnn4*Δ at 10 min intervals during the 1 h observation period. Statistical significance was evaluated using two-way analysis of variance (ANOVA) and Bonferroni Multiple Analysis Comparison Tests. * *p* < 0.05; ** *p* < 0.01; *** *p* < 0.001; 50 macrophages analysed from independent experiments (*n* = 3). All of the values were significantly different from the normalised average velocity.

Previous studies have shown that *C. albicans* phagocytosis by macrophages was altered by fungal cell wall composition [22]; however, the extent to which different stages of the phagocytic process (migration, recognition, engulfment, digestion) contributed to the observed overall effect is not known. J774.1 macrophages were exposed to four glycosylation defective *C. albicans* strains (*mnt1*Δ*mnt2*Δ, *pmr1*Δ, *mns1*Δ, and *mnn4*Δ (Table 1)), all of which have previously been shown to be phagocytosed differentially [22]. Migration track diagrams suggested enhanced macrophage migration towards the glycosylation mutants when compared to wild type macrophages (Figure S2). For every strain used in this study, migration was greater at the earlier stages of the experiment and gradually decreased over time (Figure 2). Macrophage migration was enhanced towards the *mnt1*Δ*mnt2*Δ strain (1.45 ± 0.03 SD, *p* < 0.05, 50 macrophages, *n* = 3) when compared to the wildtype CAI4 strain (1.33 ± 0.04 SD after 1 h tracking). Elevated migration was observed throughout all 10 min intervals within the 1 h observation period, but was most pronounced during the first 20 min of the experiment (CAI4 1.53 ± 0.17 SD; *mnt1*Δ*mnt2*Δ 1.69 ± 10.2 SD, *p* < 0.001; Figure 2a). No significant difference was observed for a strain functionally restored with a copy of *MNT1 mnt1*Δ*mnt2*Δ+*MNT1* (1.37 ± 0.02 SD, 1 h) when compared to CAI4 (Figure 2a).

For the other cell wall mutants assayed in this study, quantitative analysis showed no significant differences in migration towards these strains when compared to wildtype, except for *mns1*Δ (CAI4 1.39 ± 0.12 SD; *mns1*Δ 1.62 ± 0.28 SD; *p* < 0.05; Figure 2b). Nonetheless, the same trend observed for *mnt1*Δ*mnt2*Δ was identified here, with the mutants consistently exhibiting higher relative velocities than wildtype (e.g., CAI4 1.28 ± 0.06 SD, *pmr1*Δ 1.35 ± 0.06 SD, *mns1*Δ 1.44 ± 0.07 SD, and *mnn4*Δ 1.37 ± 0.15 SD, 1 h) (Figure 2b). The differences seen in migration assays for the *C. albicans* glycosylation mutants did not appear to be a consequence of increased maximum macrophage velocity (Figure S3), but a result of increased overall macrophage activity. Therefore, the glycosylation status of the target yeast cell wall had a significant effect on the migration of the macrophage towards it.

### 3.3. Macrophage Migration is Diminished within a 3D C. albicans Biofilm

**Figure 3:**
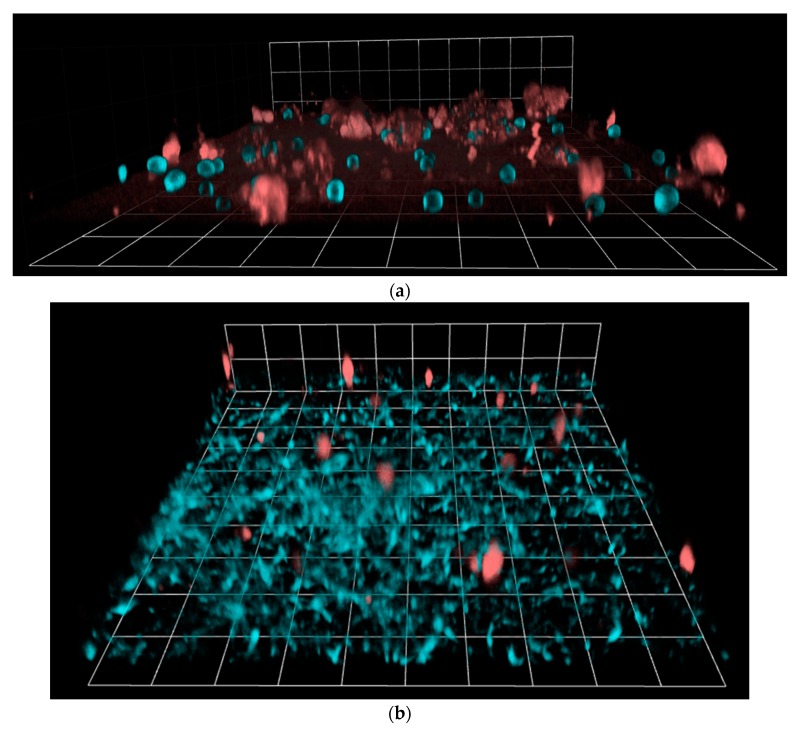

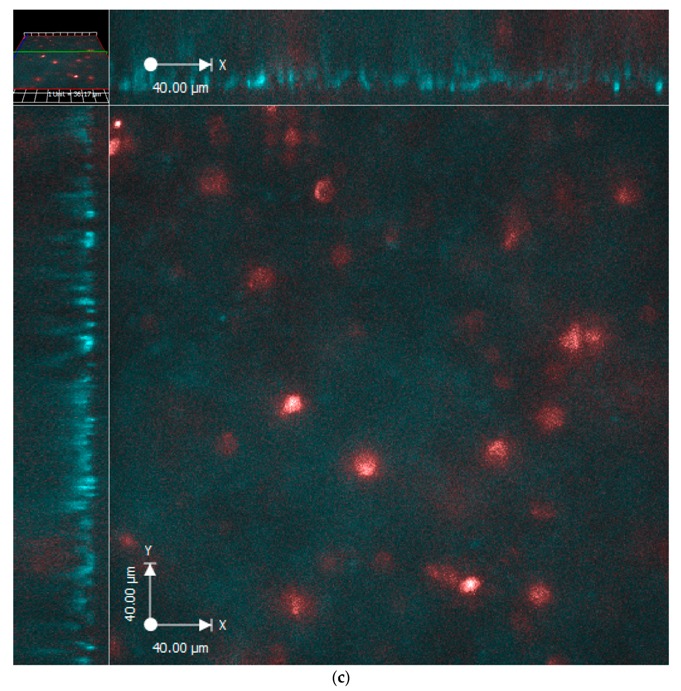
Representative single time point images acquired using Volocity software, showing J774.1 macrophages stained with LysoTracker Red (LTR; red), and *C. albicans* stained with Calcofluor White (CFW; blue). Macrophages were added in suspension to (**a**) planktonic yeast (40× objective), or (**b**) and (**c**) 48 h biofilms (20× objective); presented with (**a**) and (**b**) opacity 3D rendering, and (**c**) *x*, *y* and *z* at 20 μm depth. White grid unit or bar represents (**a**) 18.05 μm, (**b**) 36.17 μm, and (**c**) 40 μm.

Having established that a kinetic response is elicited from adherent macrophages in response to individual planktonic *C. albicans*, we next assessed phagocyte migration in response to *C. albicans* that had formed a biofilm. Typically, mature biofilms are composed of a network of yeast cells, hyphal filaments, and extracellular material that has been secreted by the fungus. Therefore, 48 h biofilms of *C. albicans* were pre-grown on imaging slides before the addition of J774.1 macrophages for 3D live imaging and analysis of macrophage migration. Because pre-adhered macrophages could not be used for experiments with *Candida* biofilms, the migration kinetics of macrophages added in suspension to either biofilms or planktonic yeast were compared. As a further control, pre-adhered macrophages responding to individual yeast were assessed. Imaging was conducted at 2 min intervals to permit sufficient time for acquiring stacks of >60 focal planes in z (Figure 3). 

**Figure 4:**
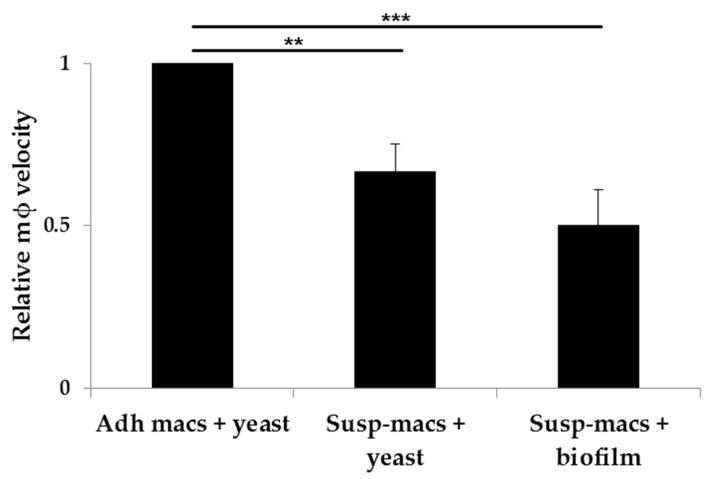
Quantitative analysis of macrophage migration in the presence of different wildtype *C. albicans*. This chart shows mean relative macrophage velocity + SD, relative to macrophage velocity of pre-adhered J774.1 (Adh-macs) responding to planktonic yeast. Relative velocities of J774.1 macrophages added in suspension (Susp-macs) to either yeast or a 48 h biofilm over a 30 min period are shown. Statistical significance was evaluated using one-way analysis of variance (ANOVA) and Tukey Multiple Analysis Comparison Tests. ** *p* < 0.01; *** *p* < 0.001; (*n* = 3).

Interactions were observed over a total of 30 min, with acquisition starting following a 45 min period to allow macrophages in suspension to descend towards the fungal targets. Macrophage migration was tracked in *x*- and *y*-axes using Volocity software. Within biofilms, migration of phagocytes was analysed at a depth of 12–20 μm from the top of the biofilm. In response to planktonic yeast, macrophages in suspension exhibited lower migratory activity, relative to adhered macrophages (0.67 ± 0.08 SD; Figure 4). Within biofilms, macrophages added into suspension exhibited severely impaired migration kinetics, relative to suspension macrophages in the presence of individual fungal cells (0.75 ± 0.12 SD), or relative to adhered macrophages in the presence of yeast (0.50 ± 0.11 SD; Figure 4). Therefore, the migration of macrophages was significantly impaired when moving through a biofilm. This was not attributed to poor migration of suspension macrophages alone, because suspension macrophages are stimulated to move significantly more in response to live fungi (Figure S4).

### 3.4. Macrophage Migration Is Diminished within 3D C. albicans Biofilms Formed from Glycosylation Defective Mutants

**Figure 5:**
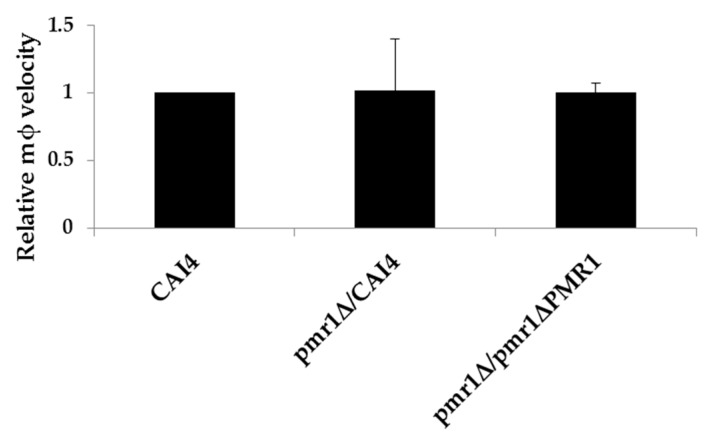
Quantitative analysis of macrophage migration within 48 h biofilms prepared from *C. albicans* strains CAI4, *pmr1*Δ, and *pmr1*Δ+*PMR1*. The chart shows mean relative macrophage velocity + SD relative to macrophage velocity of J774.1 macrophages added in suspension to CAI4 (wild type biofilms). Migration data over a 30 min period are shown. No statistical significance of mean differences was determined using one-way analysis of variance (ANOVA) and Tukey Multiple Analysis Comparison Tests.

Having established that planktonic glycosylation mutant strains of *C. albicans* elicit enhanced macrophage migration, we next sought to determine whether migration dynamics in response to biofilms depend on the glycosylation status of the fungus. An *O*-glycosylation *C. albicans* mutant lacking *MNT1* and *MNT2*, that had a single mannose modification of Ser/Thr residues in cell wall proteins, elicited the most enhanced migratory response from macrophages (Figure 2a); however, this strain was strongly impaired in its ability to establish a biofilm [23]. Recently, a strain lacking a manganese transporter encoded by *PMR1* was identified in a screen for *C. albicans* mutant strains unable to suppress neutrophil extracellular traps (NETs) [24]. The *pmr1*Δ strain that has both *O*- and *N*-linked mannan deficiencies has the capacity to form a mature biofilm [25]. We observed that the yeast cells of this mutant enhanced J774.1 macrophage migratory response (Figure 2b). Therefore, we prepared 48 h biofilms from either *pmr1*Δ or a control strain, with a single copy of *PMR1* reintegrated into the mutant (*pmr1*Δ*PMR1*) which functionally restored the wild type phenotype, [17] and determined the migration kinetics of macrophages within the biofilms. Biofilms of *pmr1*Δ were equal in thickness and no defect in hyphal growth was visible in comparison to both *pmr1*Δ*PMR1* and CAI4 biofilms. The migration kinetics of J774.1 macrophages within biofilms were determined, as described above. Although individual *C. albicans pmr1*Δ cells induced macrophage kinesis, macrophages within *pmr1*Δ biofilms exhibited the same retarded migration as in control strain biofilms (relative migration compared to CAI4 or *pmr1PMR1*, respectively: 1.02 ± 0.38, 1.01 ± 0.07 SD; Figure 5). Therefore, the mannan-deficient *pmr1*Δ biofilm did not enhance the migration capacity of macrophages, nor did planktonic *pmr1*Δ cells.

## 4. Discussion

The fungal cell surface is subject to immunosurveillance by host innate immune cells, and can be considered as a moving target, depending on the cues present at the site of *Candida* growth within various human body niches [3,4,5,6]. It has been shown previously that fungal cell wall composition affects phagocytosis of *C. albicans* by macrophages, with cell wall glycosylation mutants being engulfed to either a lesser (e.g., *mnn4*Δ and *pmr1*Δ) or greater extent (e.g., *mnt1*Δ*mnt2*Δ and *mns1*Δ) [22]. Stage-specific differences in the phagocytosis of *C. albicans*, and subsequent phagosome maturation, have been investigated in finer detail using live cell imaging [10,11,12,21].

The present study expands our understanding of the effects of fungal cell wall composition on macrophage migration in response to *C. albicans*. We show that macrophages tend to exhibit enhanced migration towards all of the glycosylation mutants tested (*mnt1*Δ*mnt2*Δ, *pmr1*Δ, *mns1*Δ and *mnn4*Δ) compared to wildtype. In all cases, migration was faster and the differences observed were greater during the earliest stages of the experiment. Whether the reduction in macrophage migration over the time course of the experiment was due to changing nutrient levels remains to be determined. The enhanced macrophage migration in response to glycosylation mutants was not a consequence of differences in maximum macrophage velocity, but of general enhanced movement. The most marked difference was observed for migration towards the *O*-linked mannan-mutant *mnt1*Δ*mnt2*Δ, confirming previous work [10]. An intermediate relative macrophage velocity was observed for macrophages attracted towards the *mnt1*Δ*mnt2*Δ+*MNT1* strain. While migration occurs in the initial stage of phagocytosis, later stages also proceed at differential rates depending on the nature of the cell wall, highlighting the importance of studying the different stages of phagocytosis separately [10,21,22]. Enhanced macrophage migration in response to *C. albicans* glycosylation cell wall mutants could be a consequence of altered wall architecture, leading to exposure of pathogen-associated molecular patterns (PAMPS) including β-glucan and chitin exposure [18], or alterations in cell wall protein distributions. Altered cell wall charge may occur, particularly for the phosphomannan-deficient strains (*mnn4*Δ and *pmr1*Δ), which could generate charge interactions to which the macrophages respond [19]. Enhanced secretion of chemoattractant molecules from *C. albicans* cell wall mutant strains may result from altered wall architecture. It is also possible that the differences in migration towards different cell wall mutants could be contact mediated if host cell filopodia were generated that were too small to be resolved in the microscopical system used. Scanning electron microscopy (SEM) images of macrophages co-cultured with *C. albicans* showed previously unseen macrophage membrane projections exceeding 40 µm [2]. Whether the presence of *Candida* with mutant cell walls drives enhanced filopod extension remains to be determined.

Macrophages infiltrate tissue during pathogen clearance and tissue homeostasis, and are thus professional migratory cells that transit within three-dimensional host tissue matrices of varying densities. Amoeboid and mesenchymal migration motions are exhibited in loose and dense tissue respectively [26]. Microbial biofilms represent an additional matrix in the host that can form on indwelling catheters and prosthetic devices. Several features of biofilms preclude therapeutic resolution and promote chronicity, including their recalcitrance to antifungal drug action, resistance to immune cells, and metabolic plasticity [27]. In the present study, we prepared *C. albicans* biofilms in vitro for 48 h to permit substantial extracellular material to be extruded. We observed that biofilms impaired the migration of macrophages by approximately 25% compared to macrophages that are added in suspension to planktonic yeast. Adherent macrophages exhibited the greatest migratory activity in response to planktonic yeast, thereby supporting the requirement for attachment to permit traction [26]. We observed that *pmr1*Δ planktonic yeast facilitated more rapid macrophage kinesis, but biofilms formed from this strain did not. The *pmr1*Δ mutant was recently identified as able to trigger NETosis by *Candida* biofilms, whereas wild type *C. albicans* biofilms block NET release [24]. The *pmr1*Δ mutant forms a mature biofilm with a lower proportion of mannan in the extracellular matrix [25], and it is the presence of a mannan-rich matrix which appears to block NETosis [24]. However, macrophage migration is significantly impaired in both wild type and *pmr1*Δ biofilms. The factors that elicit migration of macrophages in response to glycosylation mutants may not be expressed within a biofilm as this growth form is characterised by differences in protein expression [28]. Secreted compounds that may stimulate macrophage kinesis may also become sequestered in the extracellular matrix. Although the precise mechanism of biofilm suppression of macrophage migration is unclear, these findings support the hypothesis that biofilms are a further means by which pathogens evade host immune responses. Biofilms suppress fungal damage by macrophages and neutrophils [24,29,30], and may induce host responses that sustain their own growth [31]. A contiguous network of filaments and material is likely to preclude complete phagocytic enclosure of the target. Such “frustrated phagocytosis” may account for the impaired mobility of macrophages seen in *Candida* biofilms, both directly (physical restriction) and indirectly (cytokine expression). Biofilms present a multifaceted clinical problem, not only by providing a physical barrier against immune effectors or antifungal drugs, and by acting as a reservoir for seeding further infection, but as described here, by hampering phagocyte migration, thus restricting macrophage activity at such sites.

## Figures and Tables

**Table 1:** Strains of *C. albicans* used in this study.

Strain Name	Strain Number	Disrupted Target	Phenotype	Reference
CAI4-CIp10	NGY152	Not applicable (n/a)	Wild type	[13]
*mnt1*Δ/*mnt2*Δ	NGY111	α-(1,2)-mannosyltransferases	*O*-mannan defect	[14]
*mnt1*Δ/*mnt2*Δ+*MNT1*	NGY335	n/a	Complemented mutant	[14]
*pmr1*Δ	NGY355	P-type Ca^2+^/Mns^2+^-ATPase	*N*-linked mannan and *O*-linked mannan and phosphomannan defect	[15]
*pmr1*Δ+*PMR1*	NGY356	n/a	Complemented mutant	[15]
*mns1*Δ	HMY5	α-(1,2)-mannosidase	Terminal stage *N*-linked core mannan defect	[16]
*mnn4*Δ	CDH15	mannosylphosphate transferase	Phosphomannan incorporation defect	[17]

## Supplementary Materials

The following are available online at www.mdpi.com/2309-608X/3/3/31/s1.
