# Supplementary material for: Macrophage Migration Is Impaired within Candida albicans Biofilms
Source: J Fungi (Basel). 2017 Jun 22;3(3):31. doi: 10.3390/jof3030031 (PMC5715939; doi:10.3390/jof3030031)
Supplement: Supplementary file 1 [file jof-03-00031-s001.zip › Supplementary Materials.pdf]

Supplementary data

Supplementary Video S1. (.wmv) represents 60 min of interaction of J774.1 macrophages with *C. albicans* strain CAI4 at ×40 objective magnification.

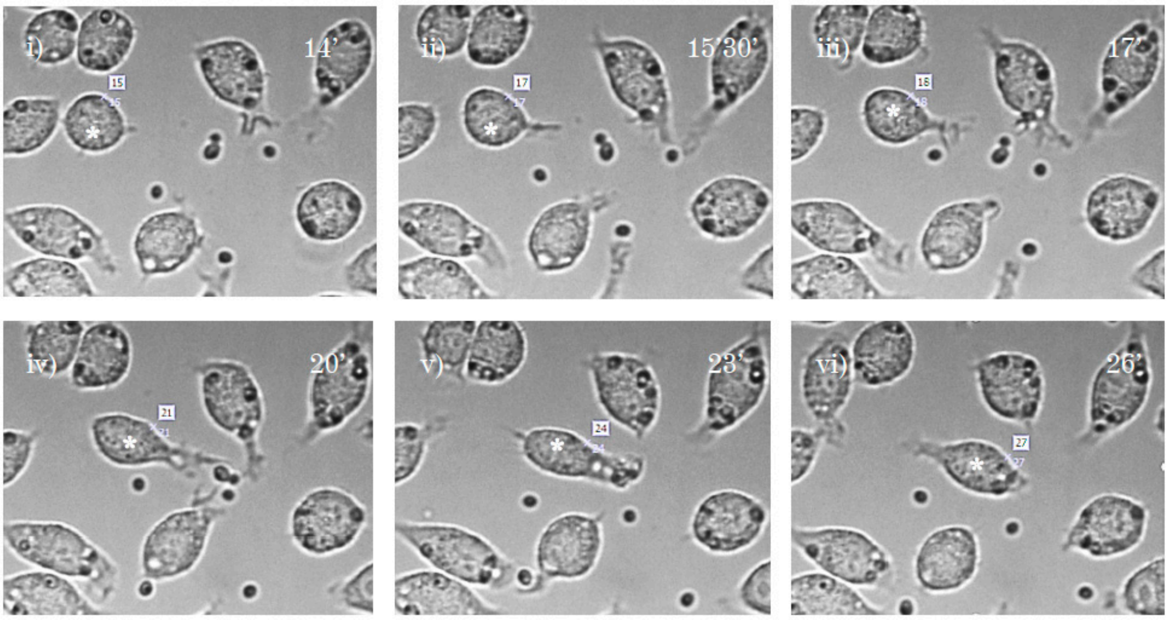

Supplementary Figure S1. Macrophage migration and engulfment of wildtype *C. albicans*. Images i-vi) are from live cell video microscopy (DIC ×20 objective) capturing the migration and engulfment processes. Image i) shows a macrophage (*) and *C. albicans* prior to cell-to-cell contact. Images ii) and iii) show macrophage polarization towards the *Candida* target. Images iv) and v) show the same cells during contact and the beginning of the engulfment process. Image vi) shows *C. albicans* within the macrophage post-engulfment. Absolute times (min) are indicated on the upper right margin of the images. White boxes reflect the manual tracking process.

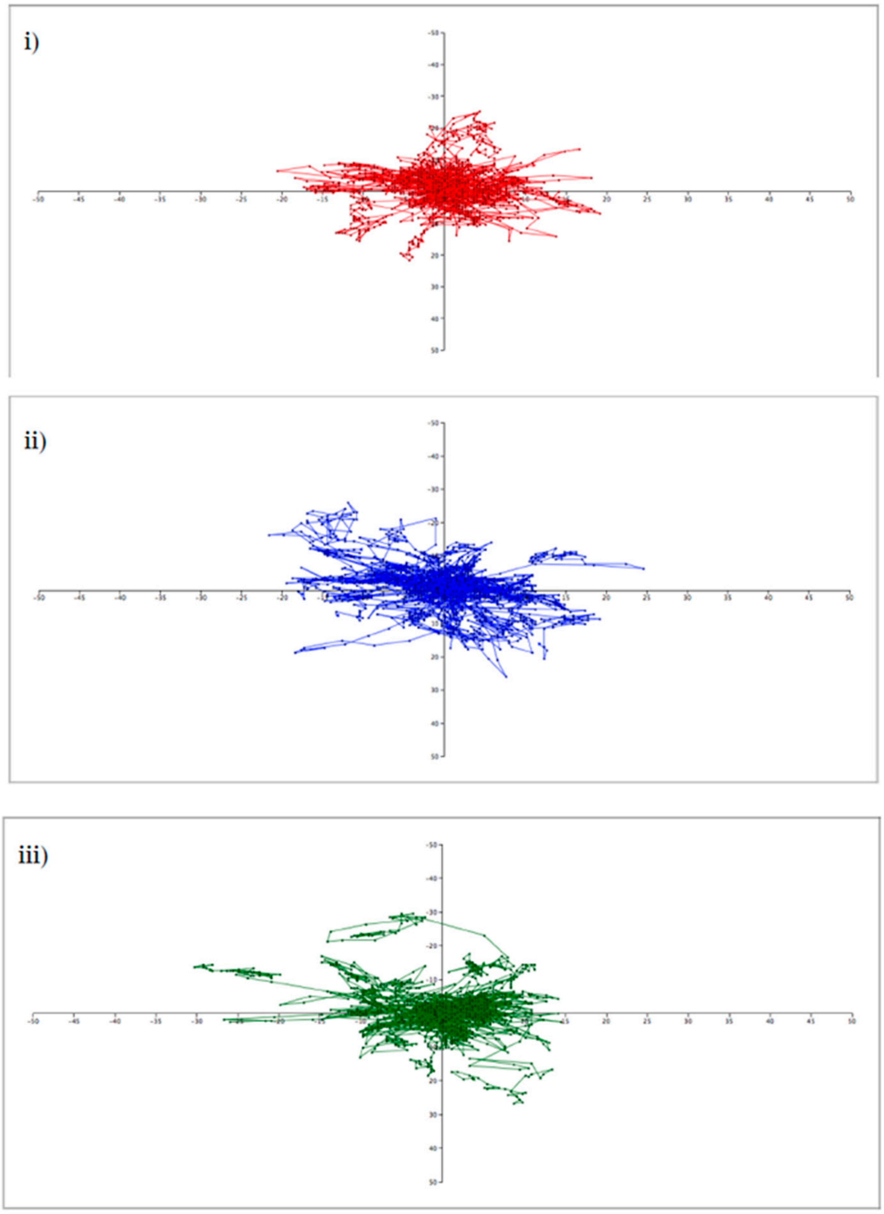

Supplementary Figure S2. Representative macrophage tracking diagrams. The tracking diagrams shown in i), ii) and iii) illustrate the distances travelled, directionality and velocity of J774.1 macrophages cultured with wildtype, *mnt1Δmnt2Δ* mutant strain and *mnt1Δmnt2Δ+MNT1* reintegrant strain, respectively. Each track represents the movement of an individual macrophage relative to its starting position, symbols indicate the location of macrophages at 90 sec intervals for 1 hour and arrows represent directionality.

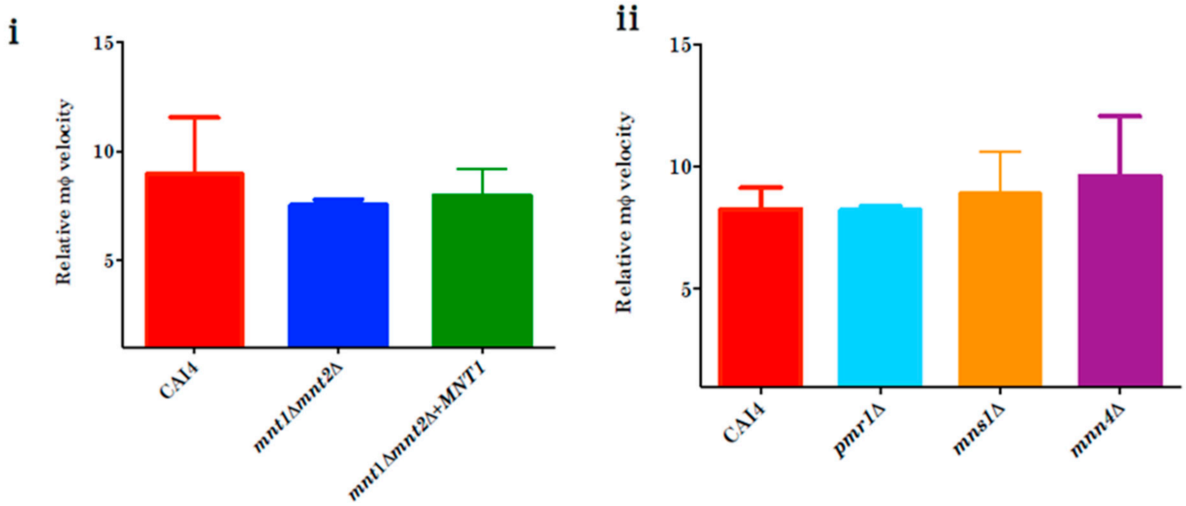

Supplementary Figure S3. Maximum relative macrophage velocities for wildtype and glycosylation mutants. The charts show mean maximum relative macrophage velocity (relative to macrophage velocity without *Candida*) + SD of J774.1 macrophages in response to i) CAI4, *mnt1*Δ*mnt2*Δ and *mnt1*Δ*mnt2*Δ+*MNT1* and ii) CAI4, pmr1Δ, mns1Δ and mnn4Δ at 1 h of analysis. Higher velocities represent instances of active macrophage chase of fungal cells. Statistical significance was evaluated using a one-way analysis of variance (ANOVA) and Tukey Multiple Analysis Comparison Tests. No statistically significant difference was observed in any of the cases (n=3).

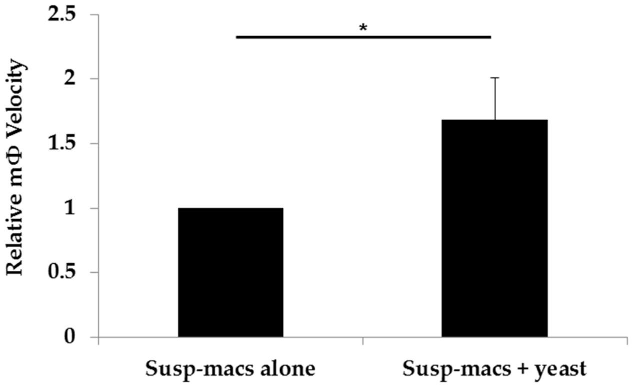

Supplementary Figure S4. Relative migration of suspension macrophages is increased in response to live fungi. The charts show mean relative macrophage velocity +SD in the presence of CAI4 relative to macrophage velocity of J774.1 macrophages over a 30 min period. Statistical significance was evaluated using Student's t-test: *, $p<0.05$.
